# Microbiological profile of infectious keratitis in the Newcastle and Gateshead region: a 10-year analysis

**DOI:** 10.1038/s41433-023-02763-x

**Published:** 2023-10-17

**Authors:** Nikolaos Tzoumas, Ana Luiza Mylla Boso, Melissa Gough, Jaswant Sandhu, Manjusha Narayanan

**Affiliations:** 1 Biosciences Institute, Newcastle University, Newcastle upon Tyne, UK; 2 Sunderland Eye Infirmary, Sunderland, UK; 3 Newcastle Eye Centre, Royal Victoria Infirmary, Newcastle upon Tyne, UK; 4 Microbiology and Virology Services, Royal Victoria Infirmary, Newcastle upon Tyne, UK

**Keywords:** Risk factors, Corneal diseases, Infection

## Introduction

Infectious keratitis is a leading cause of blindness and hospitalisation worldwide, incurring £2,855 per admission [1]. Causative organisms demonstrate marked geographical and temporal variation, and there are limited epidemiological data necessary to establish disease trends [2]. We analysed infectious keratitis patterns and trends in a regional context, informing prevention and management strategies.

## Methods

We audited corneal scrapes from a major UK NHS trust, serving Newcastle and Gateshead’s densely populated and deprived areas (population: 556,181) [3], from January 2012 to 2023. Sight-threatening corneal ulcers meeting local criteria underwent urgent investigation. Cultures were incubated for at least a week, longer for suspected *Acanthamoeba*. Microbial trends were analysed using age-adjusted Poisson regression. We considered only the first non-commensal microbial agent each admission (±30 days) to avoid duplications.

## Results

We found 600 culture-positive cases involving 72 species and 31 genera; 10% were repeat cases. Bacteria comprised 88.8%, fungi 7.0%, and *Acanthamoeba* 4.2%. Positive scrape yield averaged 37.9% per admission. Median age was 59 (IQR 42–75) years, stable throughout.

**Fig. 1:**
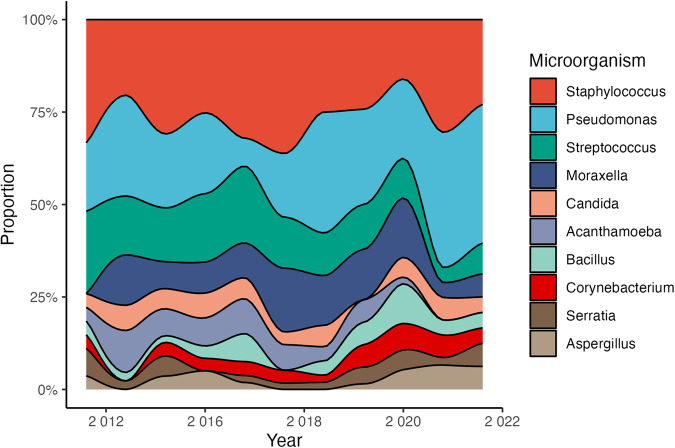
Area plot showing the trends of the most prevalent infectious keratitis pathogens over the past decade. Each line represents the cumulative proportion of culture-positive cases for that year. The relative incidence of *Staphylococcus* has remained stable, while *Pseudomonas* has shown an upward trend in recent years.

The commonest genera were *Staphylococcus* (25%), *Pseudomonas* (22%), *Streptococcus* (12%), and *Moraxella* (10%). Gram-positive and negative representation was equal (44%, 43%). Although *Staphylococcus* cases remained stable (0% annual change, *P* = 0.99), *Pseudomonas* cases rose significantly (+7%, *P* = 0.003), establishing it as the predominant infectious agent over the past four years (Fig. 1). *Pseudomonas* keratitis peaked July to November (27–42%) *vs*. rest of year (5–20%). There were no consistent changes in other pathogens’ rates, including *Acanthamoeba* (−8%, *P* = 0.34; Supplementals).

Peak months were May, July, August, September, and October (9–10% each). The busiest period was the last week of June and the first week of July (Supplementals). Gram-positive pathogens prevailed in May (57%) and January (55%), while Gram-negative pathogens predominated in November (58%) and August (55%). On weekdays, most samples were processed Tuesdays (21%) and Saturdays (18%). These timings do not consider potential delays in sample handling.

## Discussion

We reveal a sustained increase in *Pseudomonas*-related keratitis rates across Northern England, recently surpassing *Staphylococcus* cases (Fig. 1). This aligns with other UK regions’ trend of declining Gram-positive infections, favouring Gram-negative pathogens like *Pseudomonas* [4, 5]. This shift may reflect changing contact lens use behaviours, a recognised risk for Gram-negative infections. However, it remains unclear why this practice would not have led to a similar trend in *Acanthamoeba* keratitis – this disparity could stem from softer water compared to the South [6].

We also confirm bacteria as the main cause of infectious keratitis, comprising 89% of culture-positive cases (previously reported: 91–93%) [2]. *Staphylococcus* and *Pseudomonas* dominated our sample (25%, 22%), surpassing rates in other UK areas like neighbouring Sunderland (14%, 12%) [2]. Non-bacterial keratitis rates were comparable [2], hinting at limited microbial diversity possibly due to elevated antibiotic use [7]. Additionally, we confirmed increased culture-positive cases in warmer months [5], and identified increased rates on Tuesdays and Saturdays, likely due to higher patient visits to our Eye Casualty the previous day [3].

Further epidemiological study is urgently needed to establish UK-wide infectious keratitis trends and identify preventable causal relationships.

### Supplementary information

Supplementary Information

## Data Availability

Summative data generated or analysed during this study are included in this published article and its supplementary information. Further data available on request.
