# Supplementary material for: Microbiological profile of infectious keratitis in the Newcastle and Gateshead region: a 10-year analysis
Source: Eye (Lond). 2023 Oct 17;38(4):813–4. doi: 10.1038/s41433-023-02763-x (PMC10920867; doi:10.1038/s41433-023-02763-x)

**Supplementary Information**

**Supplementary Table.** Incidence of most common infectious keratitis microorganisms over the study period. Smoothed conditional means are plotted, and p-values indicate the results of age-adjusted Poisson regression estimates.

| Genus | Year | n |  | *P* |
| --- | --- | --- | --- | --- |
| *Acanthamoeba* | 2012 | 1 | ***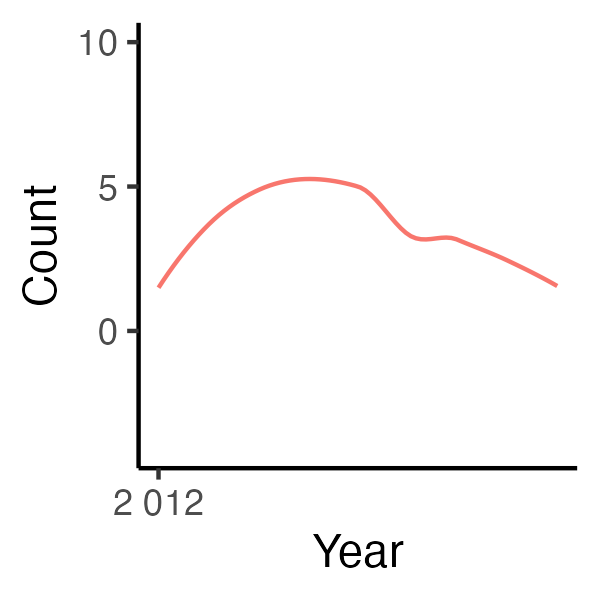*** | 0.34 |
|  | 2013 | 5 |  |  |
|  | 2014 | 4 |  |  |
|  | 2016 | 5 |  |  |
|  | 2017 | 4 |  |  |
|  | 2018 | 2 |  |  |
|  | 2019 | 4 |  |  |
|  | 2020 | 1 |  |  |
| *Aspergillus* | 2012 | 1 | ***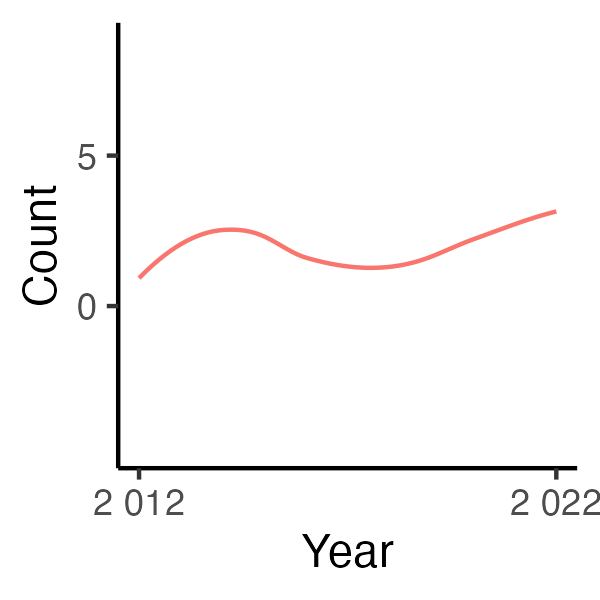*** | 0.39 |
|  | 2014 | 2 |  |  |
|  | 2015 | 3 |  |  |
|  | 2016 | 1 |  |  |
|  | 2019 | 1 |  |  |
|  | 2020 | 3 |  |  |
|  | 2022 | 3 |  |  |
| *Bacillus* | 2012 | 1 | ***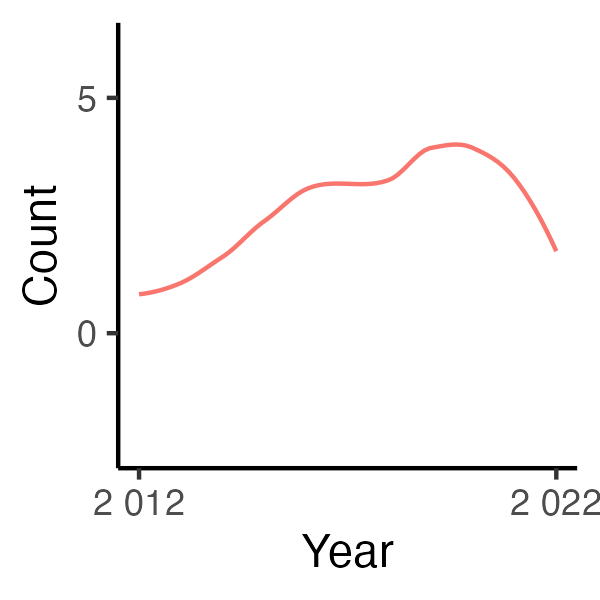*** | 0.13 |
|  | 2013 | 1 |  |  |
|  | 2014 | 1 |  |  |
|  | 2015 | 2 |  |  |
|  | 2016 | 4 |  |  |
|  | 2018 | 2 |  |  |
|  | 2019 | 4 |  |  |
|  | 2020 | 6 |  |  |
|  | 2021 | 2 |  |  |
|  | 2022 | 2 |  |  |
| *Candida* | 2012 | 1 | ***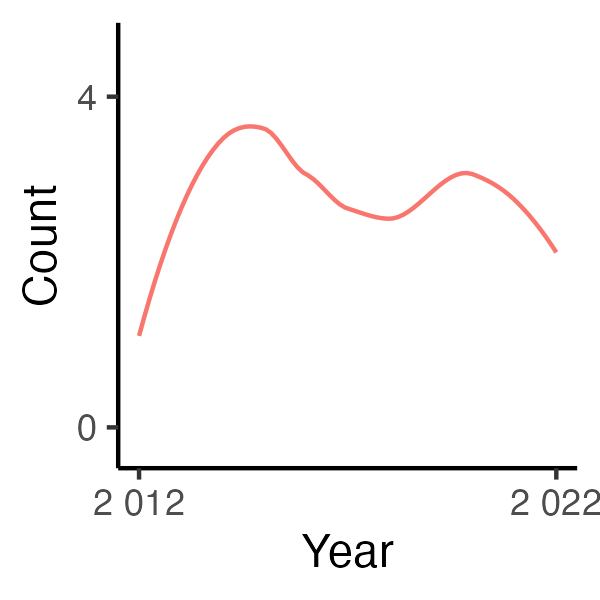*** | 0.97 |
|  | 2013 | 3 |  |  |
|  | 2014 | 3 |  |  |
|  | 2015 | 4 |  |  |
|  | 2016 | 3 |  |  |
|  | 2017 | 2 |  |  |
|  | 2018 | 3 |  |  |
|  | 2020 | 3 |  |  |
|  | 2021 | 3 |  |  |
|  | 2022 | 2 |  |  |
| *Corynebacterium* | 2012 | 1 | ***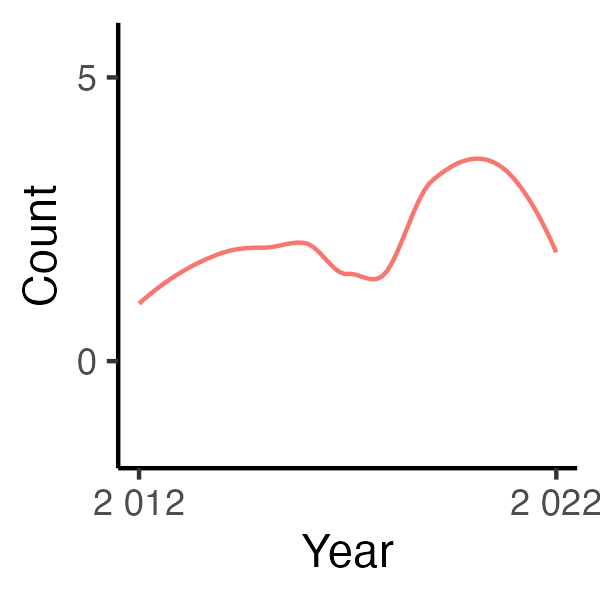*** | 0.38 |
|  | 2014 | 2 |  |  |
|  | 2015 | 2 |  |  |
|  | 2016 | 2 |  |  |
|  | 2017 | 2 |  |  |
|  | 2018 | 1 |  |  |
|  | 2019 | 4 |  |  |
|  | 2021 | 3 |  |  |
|  | 2022 | 2 |  |  |
| *Moraxella* | 2013 | 6 | ***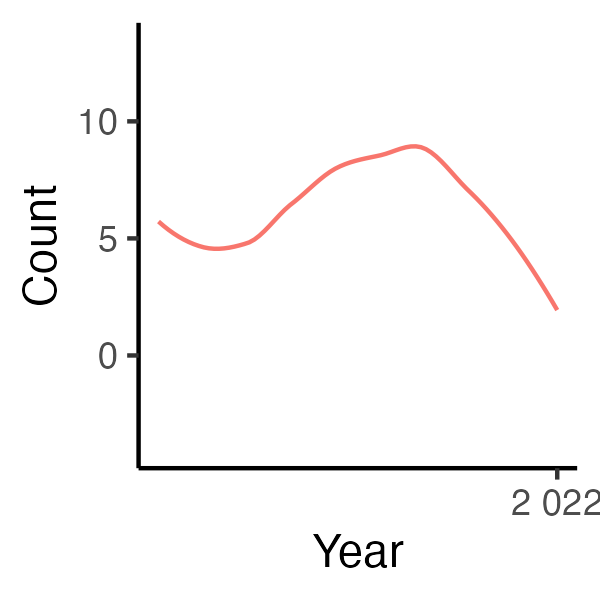*** | 0.55 |
|  | 2014 | 4 |  |  |
|  | 2015 | 5 |  |  |
|  | 2016 | 5 |  |  |
|  | 2017 | 10 |  |  |
|  | 2018 | 7 |  |  |
|  | 2019 | 9 |  |  |
|  | 2020 | 9 |  |  |
|  | 2021 | 2 |  |  |
|  | 2022 | 3 |  |  |
| *Pseudomonas* | 2012 | 5 | ***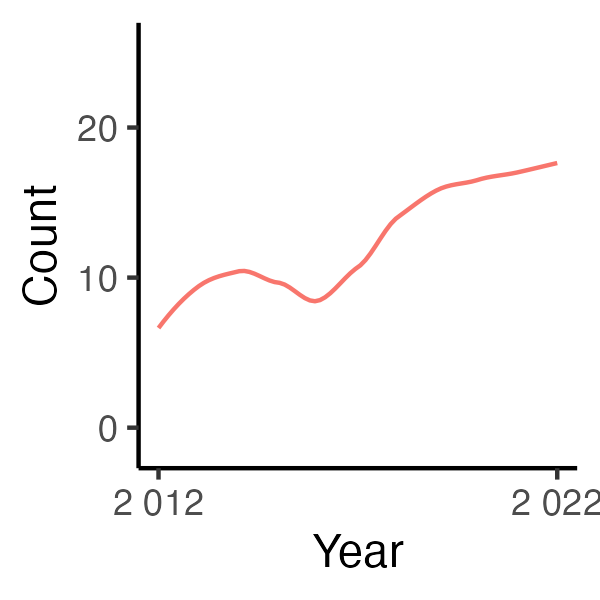*** | 0.003 |
|  | 2013 | 12 |  |  |
|  | 2014 | 11 |  |  |
|  | 2015 | 13 |  |  |
|  | 2016 | 4 |  |  |
|  | 2017 | 10 |  |  |
|  | 2018 | 17 |  |  |
|  | 2019 | 17 |  |  |
|  | 2020 | 12 |  |  |
|  | 2021 | 18 |  |  |
|  | 2022 | 18 |  |  |
| *Serratia* | 2012 | 2 | ***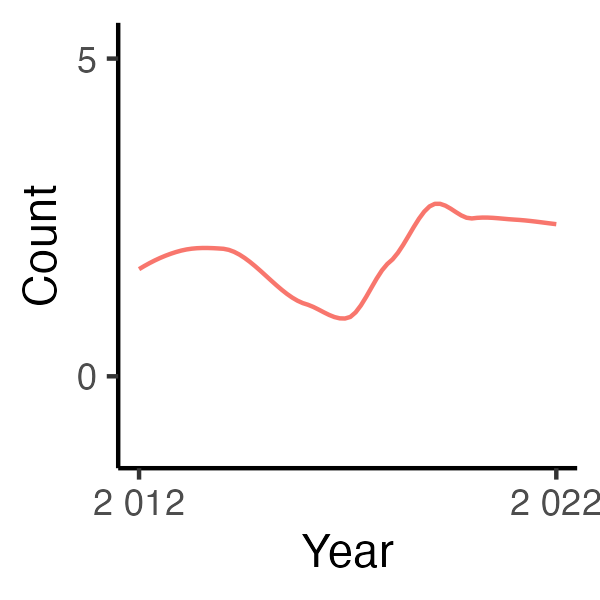*** | 0.52 |
|  | 2013 | 1 |  |  |
|  | 2014 | 3 |  |  |
|  | 2016 | 1 |  |  |
|  | 2017 | 1 |  |  |
|  | 2018 | 1 |  |  |
|  | 2019 | 3 |  |  |
|  | 2020 | 3 |  |  |
|  | 2021 | 1 |  |  |
|  | 2022 | 3 |  |  |
| *Staphylococcus* | 2012 | 9 | ***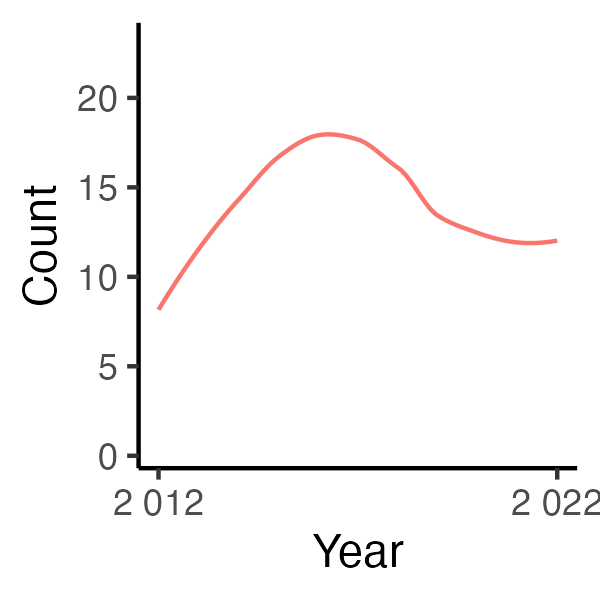*** | 0.99 |
|  | 2013 | 9 |  |  |
|  | 2014 | 17 |  |  |
|  | 2015 | 15 |  |  |
|  | 2016 | 17 |  |  |
|  | 2017 | 21 |  |  |
|  | 2018 | 13 |  |  |
|  | 2019 | 16 |  |  |
|  | 2020 | 9 |  |  |
|  | 2021 | 15 |  |  |
|  | 2022 | 11 |  |  |
| *Streptococcus* | 2012 | 6 | ***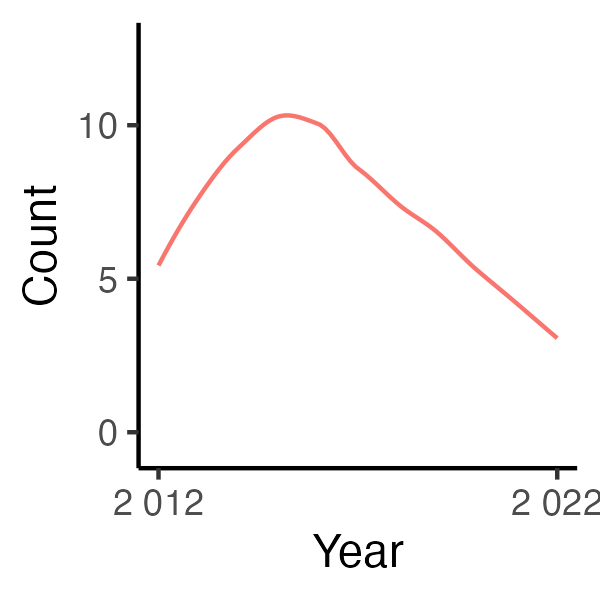*** | 0.06 |
|  | 2013 | 7 |  |  |
|  | 2014 | 8 |  |  |
|  | 2015 | 11 |  |  |
|  | 2016 | 11 |  |  |
|  | 2017 | 8 |  |  |
|  | 2018 | 6 |  |  |
|  | 2019 | 8 |  |  |
|  | 2020 | 6 |  |  |
|  | 2021 | 2 |  |  |
|  | 2022 | 4 |  |  |

**Supplementary Figure 1.** Yearly culture-positive corneal scrape results by microorganism.


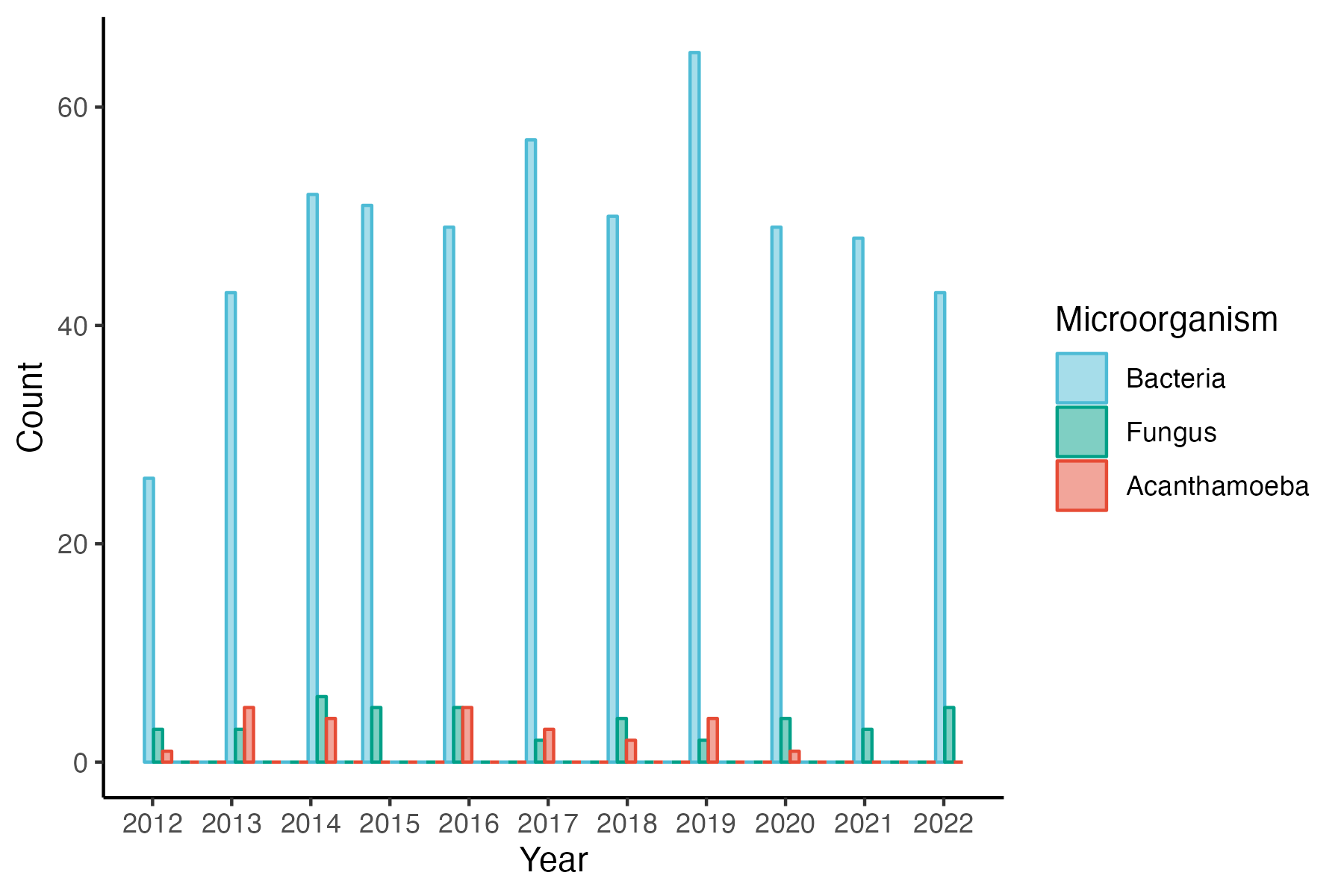


**Supplementary Figure 2.** Monthly culture-positive corneal scrape results by microorganism.


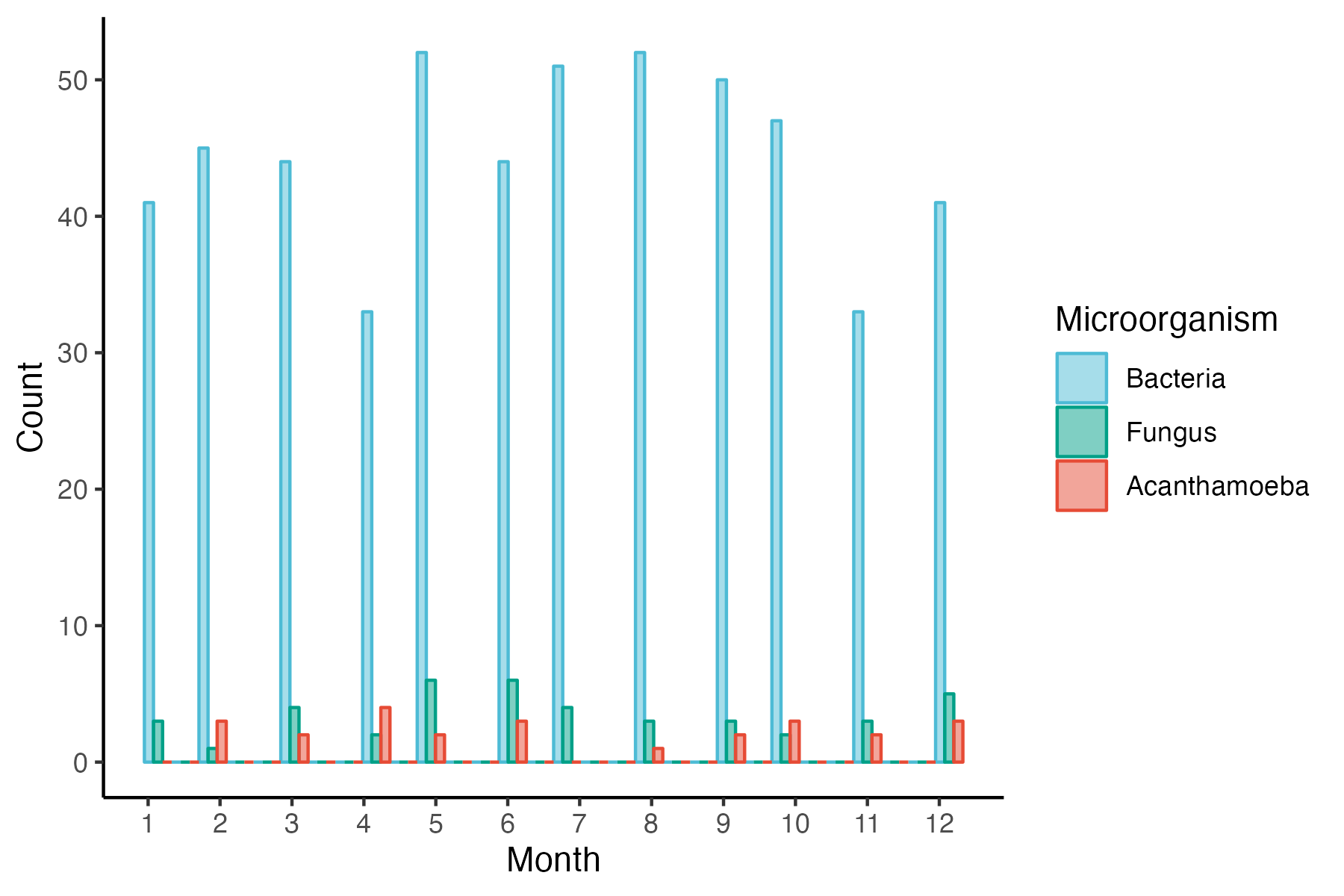


**Supplementary Figure 3.** Monthly culture-positive corneal scrape results by Gram stain.


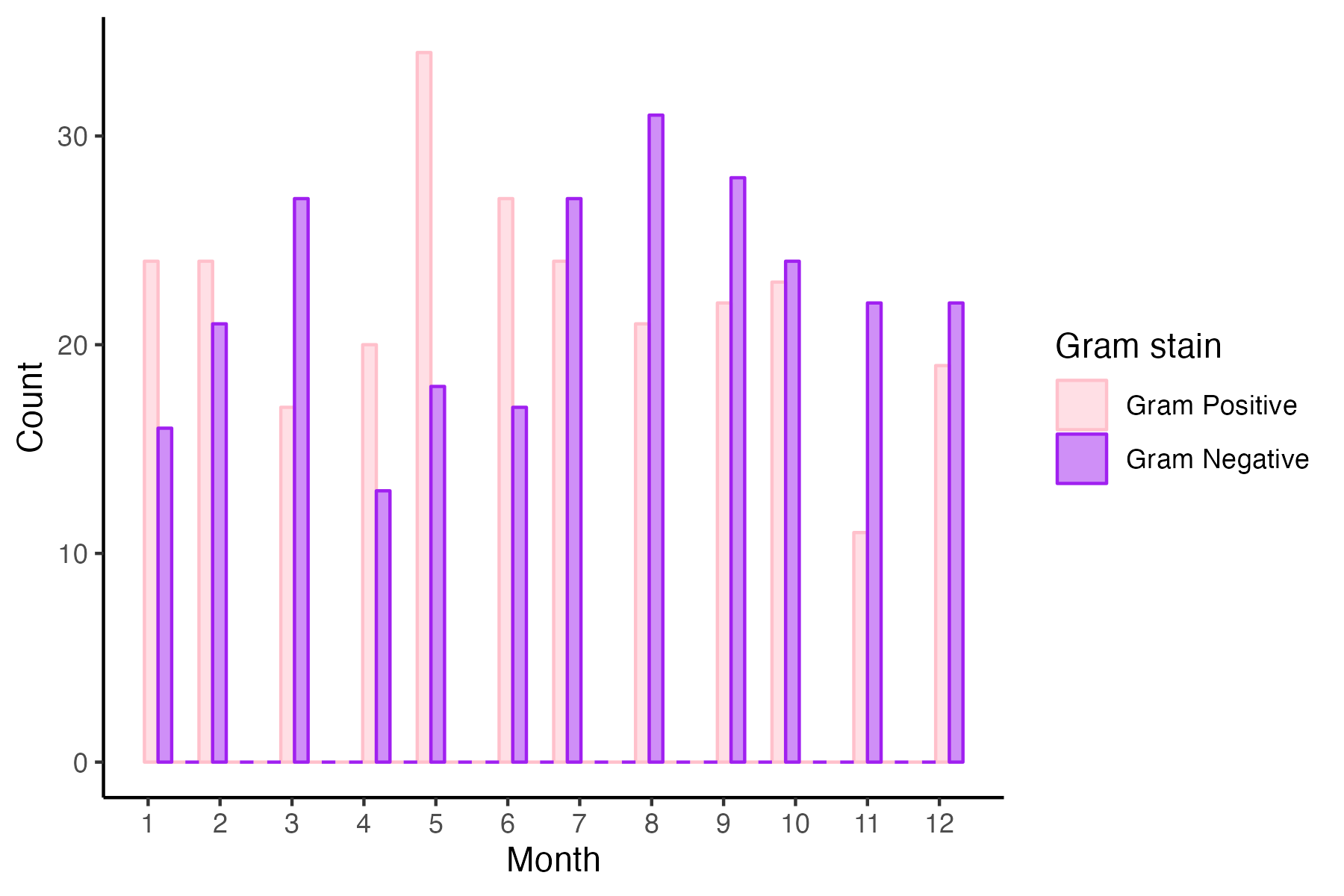


**Supplementary Figure 4.** Weekly culture-positive corneal scrape results by microorganism.


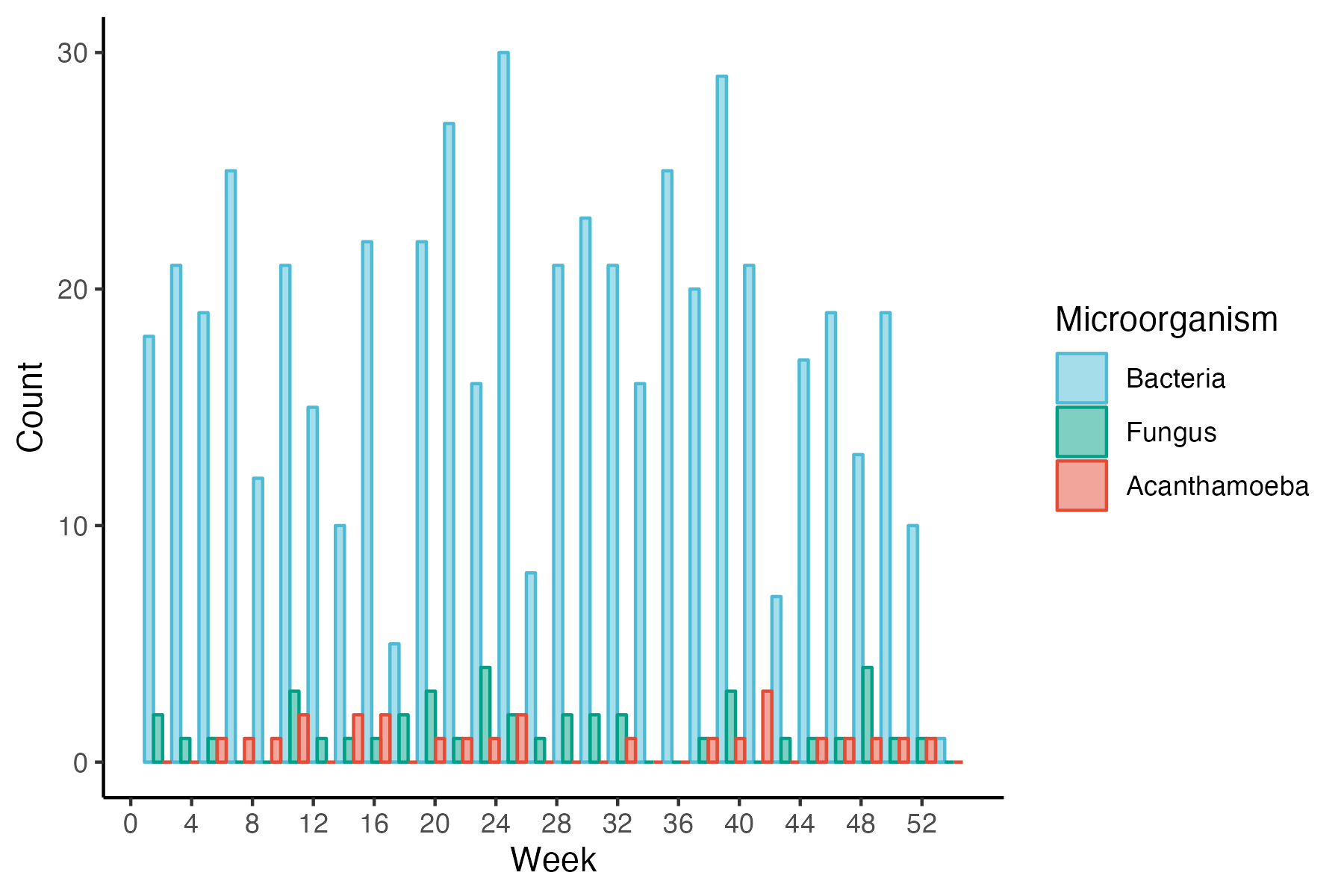


**Supplementary Figure 5.** Weekday culture-positive corneal scrape results by microorganism.


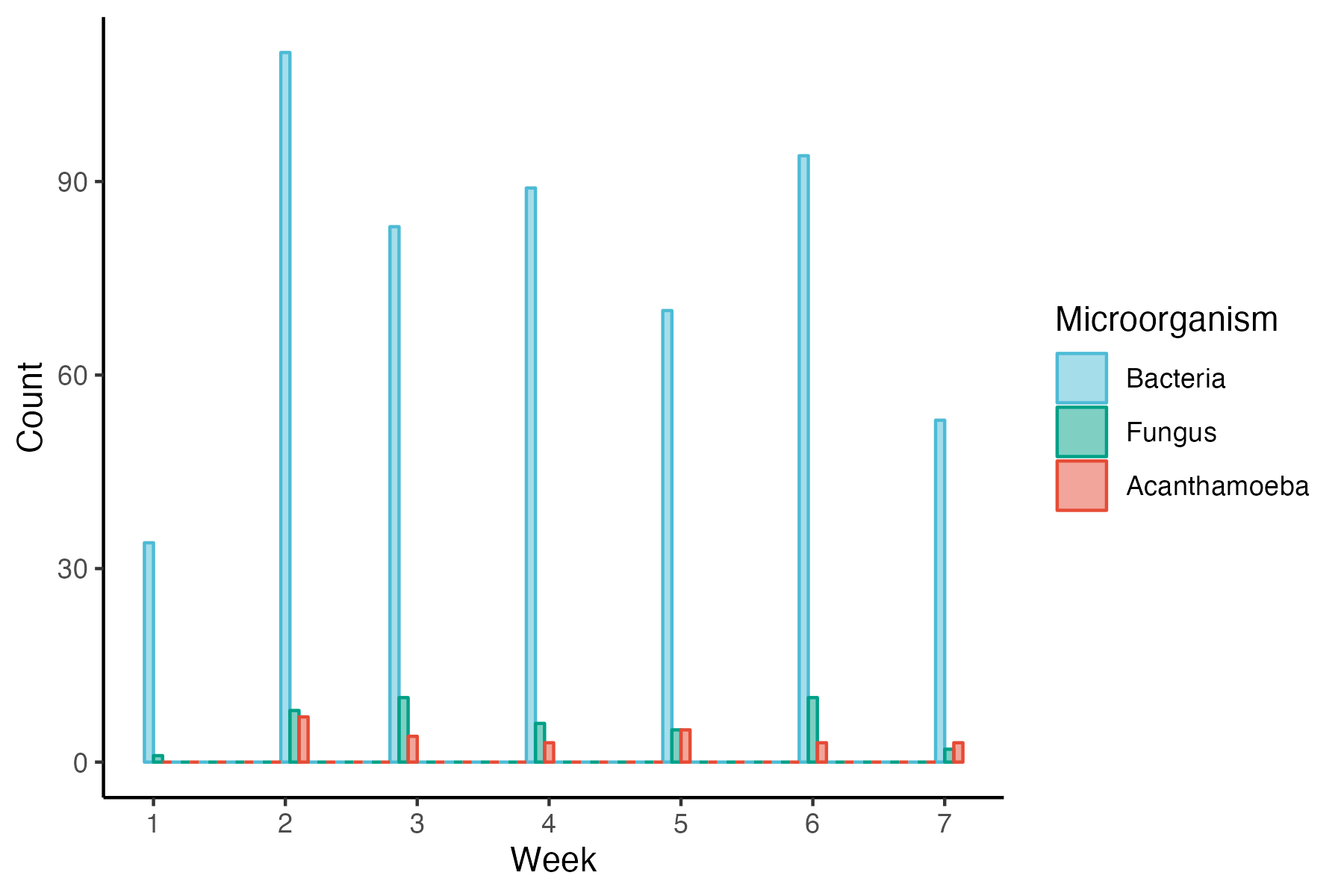

Supplement: Supplementary file 1 — Supplementary Information [file 41433_2023_2763_MOESM1_ESM.docx]
